# Estimating Individual-Level Exposure to Airborne Polycyclic Aromatic Hydrocarbons throughout the Gestational Period Based on Personal, Indoor, and Outdoor Monitoring

**DOI:** 10.1289/ehp.10972

**Published:** 2008-07-16

**Authors:** Hyunok Choi, Frederica Perera, Agnieszka Pac, Lu Wang, Elzbieta Flak, Elzbieta Mroz, Ryszard Jacek, Tricia Chai-Onn, Wieslaw Jedrychowski, Elizabeth Masters, David Camann, John Spengler

**Affiliations:** 1 Columbia Center for Children’s Environmental Health, Mailman School of Public Health, Columbia University, New York, New York, USA; 2 Epidemiology and Preventive Medicine, College of Medicine, Jagiellonian University, Krakow, Poland; 3 Department of Biostatistics, Harvard School of Public Health, Boston, Massachusetts, USA; 4 Center for International Earth Science Information Network, Columbia University, Palisades, New York, USA; 5 Department of Analytical and Environmental Chemistry, Southwest Research Institute, San Antonio, Texas, USA; 6 Department of Environmental Health, Harvard School of Public Health, Boston, Massachusetts, USA

**Keywords:** coal, long-term personal exposure, polycyclic aromatic hydrocarbons, spatial and temporal variability

## Abstract

**Objectives:**

Current understanding on health effects of long-term polycyclic aromatic hydrocarbon (PAH) exposure is limited by lack of data on time-varying nature of the pollutants at an individual level. In a cohort of pregnant women in Krakow, Poland, we examined the contribution of temporal, spatial, and behavioral factors to prenatal exposure to airborne PAHs within each trimester and developed a predictive model of PAH exposure over the entire gestational period.

**Methods:**

We monitored nonsmoking pregnant women (*n* = 341) for their personal exposure to pyrene and eight carcinogenic PAHs—benz[*a*]anthracene, chrysene/isochrysene, benzo[*b*]fluoranthene, benzo[*k*]fluoranthene, benzo[*a*]pyrene [B(*a*)P], indeno[*1,2,3*-*c*,*d*]pyrene, dibenz[*a*,*h*]anthracene, and benzo[*g*,*h*,*i*]perylene—during their second trimester for a consecutive 48-hr period. In a subset (*n* = 78), we monitored indoor and outdoor levels simultaneously with the personal monitoring during the second trimester with an identical monitor. The subset of women was also monitored for personal exposure for a 48-hr period during each trimester. We repeatedly administered a questionnaire on health history, lifestyle, and home environment.

**Results:**

The observed personal, indoor, and outdoor B(*a*)P levels we observed in Krakow far exceed the recommended Swedish guideline value for B(*a*)P of 0.1 ng/m^3^. Based on simultaneously monitored levels, the outdoor PAH level alone accounts for 93% of total variability in personal exposure during the heating season. Living near the Krakow bus depot, a crossroad, and the city center and time spent outdoors or commuting were not associated with higher personal exposure. During the nonheating season only, a 1-hr increase in environmental tobacco smoke (ETS) exposure was associated with a 10–16% increase in personal exposure to the nine measured PAHs. A 1°C decrease in ambient temperature was associated with a 3–5% increase in exposure to benz[*a*]anthracene, benzo[*k*]fluoranthene, and dibenz[*a*,*h*]anthracene, after accounting for the outdoor concentration. A random effects model demonstrated that mean personal exposure at a given gestational period depends on the season, residence location, and ETS.

**Conclusion:**

Considering that most women reported spending < 3 hr/day outdoors, most women in the study were exposed to outdoor-originating PAHs within the indoor setting. Cross-sectional, longitudinal monitoring supplemented with questionnaire data allowed development of a gestation-length model of individual-level exposure with high precision and validity. These results are generalizable to other nonsmoking pregnant women in similar exposure settings and support reduction of exposure to protect the developing fetus.

Polycyclic aromatic hydrocarbons (PAHs) are globally distributed compounds associated with anthropogenic combustion and/or pyrolysis of fossil fuel, industrial or domestic coal, wood, cigarettes, and food items (Bostrom et al. 2002). Although local generation is an important exposure source, long-range transport and volatilization of deposited PAHs also contribute to urban ambient concentrations (Dimashki et al. 2001).

Long-term exposure to PAHs has been associated with increased risks of cardiopulmonary mortality (Martin et al. 2007; Pei et al. 2002) and lung cancer mortality (Hoshuyama et al. 2006; Matsumoto et al. 2007), as well as developmental toxicity (Choi et al. 2006; Hoshuyama et al. 2006; Perera et al. 2002, 2003; Yu et al. 2006). Traditional epidemiologic approaches to assessing long-term effects of air pollution exposure often rely on ambient exposure data, individual-level health outcomes, and individual-level confounder variables. Estimation of personal exposure to air pollutants using ambient monitoring data suffers from the potential for exposure misclassification due to intraurban variability in outdoor concentrations of the pollutants (Briggs et al. 2000), as well as the variability in personal behavioral patterns, such as exposure to environmental tobacco smoke (ETS) (Georgiadis et al. 2001). In such semiecologic designs, air pollution risk parameters quantify group-level risk rather than individual-level risk (Haneuse et al. 2007). Ignoring the time-varying nature as well as small-area variation in air pollution exposure could result in biased estimates of effects (Haneuse et al. 2007). Furthermore, in many epidemiologic investigations of outcomes with long latency periods, the mean exposure concentration for a multiyear period is assumed to approximate personal exposure. Because not only the exposure but also host susceptibility may change over time, such an assumption could further contribute to a biased estimate of true risk.

Several studies estimated spatial distribution of airborne PAHs from local sources in terms of annual mean benzo[*a*]pyrene [B(*a*)P] equivalent concentrations (Nielsen et al. 1996; Tao et al. 2006). However, to date, no study has examined an inhalation exposure to PAHs for an extended period of time based on direct monitoring of the individual. As a result, little is known about the validity and precision of long-term (i.e., several years to lifetime) PAH exposure estimation at an individual level. In settings where there is small seasonal variation and no long-term temporal trend in air pollution, long-term exposure to particulate matter with aerodynamic diameters ≤ 2.5 μm (PM_2.5_) can be estimated (Wallace and Williams 2005). However, application of this method to other populations is limited because of dissimilar sources, meteorologic conditions, and other social and cultural factors. For example, personal exposure to the sum of eight carcinogenic PAHs (∑8c-PAHs) in Krakow, Poland, varies by > 10-fold across seasons (Choi et al. 2006). In addition, mean personal exposure to PM_2.5_ for women monitored during the heating season was significantly higher than for women monitored during the nonheating season (43.37 vs. 29.77 μg/m^3^, *p* < 0.001) (Jedrychowski et al. 2006).

Another important consideration in long-term exposure estimation is the particular health outcome of interest. There is considerable evidence that host susceptibility associated with age of exposure is critical in determining the severity of adverse outcomes (Dejmek et al. 2000; Sanyal et al. 1993; Wormley et al. 2004). For example, effects of B(*a*)P on embryo trophoblasts (Sanyal et al. 1993) have been shown to vary depending on the period of exposure as well as the exposed organ (Sanyal et al. 1993; Wormley et al. 2004). Varying fetal vulnerability across the gestational period is caused not only by the changes in rate of cell division, migration, differentiation, and apoptosis, but also by the immaturity of the detoxification mechanisms and DNA repair capabilities (Anderson et al. 2000; Selevan et al. 2000).

The present analysis is part of an ongoing prospective cohort study on prenatal and early childhood exposure to multiple toxicants on a number of developmental and health outcomes (Choi et al. 2006; Jedrychowski et al. 2004, 2005a, 2005b, 2006). Here, we focus on developing a gestation-length model of personal exposure to ∑8c-PAH among pregnant women enrolled in an ongoing study in Krakow. Because of their high mutual correlations, we assumed in our previous analyses that ∑8c-PAHs represents the combined fetotoxicity of the eight carcinogenic PAHs monitored in the present study. We examined the validity of this assumption by observing whether the personal exposure patterns to each of the eight individual PAHs are consistent with those for ∑8c-PAHs. The objectives of this analysis were *a*) to characterize PAH exposure based on simultaneous personal, home indoor, and home outdoor monitoring; *b*) to identify short-term risk factors of personal exposure; and *c*) to build a predictive model for mean individual-level exposure during each month of gestation, based on these results.

## Materials and Methods

Details regarding subject enrollment and air monitoring methods have been previously published (Choi et al. 2006; Jedrychowski et al. 2004, 2006) and can also be found in our Supplemental Material (online at http://www.ehponline.org/members/2008/10972/suppl.pdf). Briefly, we recruited nonsmoking pregnant women from the prenatal care clinics from areas with well-recorded ambient air pollution levels in the Srodmiescie–Old Podgorze area (city center) and the Krowodrza–Nowa Huta–New Podgorze area (city outer area) (Jedrychowski et al. 2003). We chose the enrollment target sites based on 1996 air monitoring data from the U.S. Environmental Protection Agency (EPA), which showed that B(*a*)P levels were 2-fold higher in Srodmiescie (annual mean, 13.3 ng/m^3^; 22.4 ng/m^3^ in winter; 4.6 ng/m^3^ in summer) than in Krowodrza (annual mean, 6 ng/m^3^; 11.2 ng/m^3^ in winter; 0.9 ng/m^3^ in summer). To capture seasonal variability in personal exposure, we recruited approximately equal proportions of the total cohort during each season (23% enrolled during December–February; 27%, during March–May; 26% during June–August; and 24% during September–November).

### Personal interview

A research staff member administered a questionnaire to the women in their homes during their late second trimester. The questionnaire elicited information on demographic, health history, socioeconomic profile, outdoor environment, housing characteristics, indoor exposure sources, and other lifestyle choices. We restricted our analysis of the questionnaire data to the factors that potentially contribute to the exposure to PAHs, including smoking history by household members and dietary intake of PAH-containing foods (Table 1). Because behavior patterns might change through the pregnancy course, we administered the questionnaires again during the third trimester.

### Personal PAH monitoring

We measured personal exposure directly at the individual level by having each pregnant woman wear a backpack in all microenvironments during waking hours and place it at bedside at night, for a consecutive 48-hr period [see Supplemental Material (online at http://www.ehponline.org/members/2008/10972/suppl.pdf)]. Because we did not ask the subjects to keep a log of the time spent in each microenvironment, we interpreted personal exposures as the cumulative concentration over the 48-hr monitoring period. In a randomly assigned subset of 78 women, we conducted repeat personal monitoring for a 48-hr period during each trimester.

### Indoor and outdoor air monitoring

In the subset of 78 women, we conducted indoor and outdoor PAH monitoring at the same time as the second-trimester personal monitoring. The indoor samplers were placed in a room where the woman spent most of her time (i.e., living room or near the kitchen). The sampler was placed atop furniture 0.5–2 m above the floor away from the heating sources, about 1 m away from the wall of the home or the apartment. All sampling units were checked and batteries replaced by staff approximately midway through the 48-hr period.

### Laboratory analysis of PAHs in air monitoring samples

We removed the quartz filter and polyurethane foam (PUF) plug, spiked with *p*-terphenyl-d_14_ (as an extraction surrogate), and performed Soxhlet extraction (Corning Inc., Corning, NY, USA) with 6% diethyl ether in hexanes for 16 hr. Each extract was concentrated to a final volume of 1.0 mL and frozen at −12°C. We analyzed the air extracts by gas chromatography/mass spectrometry for pyrene and the eight carcinogenic PAHs—benz[*a*]anthracene [B(*a*)A], chrysene/isochrysene, benzo[*b*]fluoranthene [B(*b*)F], benzo[*k*]fluoranthene [B(*k*)F], B(*a*)P, indeno[*1,2,3-c*,*d*]pyrene, dibenz[*a*,*h*]anthracene [D(*a*,*h*)A], and benzo[*g*,*h*,*i*]perylene [B(*g*,*h*,*i*)P]—as described in U.S. EPA (1999). The recovery of the extraction surrogate, *p*-terphenyl-d_14_, was consistently > 60%, indicating satisfactory recovery of collected PAHs. The 48 matrix spikes showed that the procedure efficiently extracted all nine PAHs from the filter and PUF, with recovery means ranging from 91% to 117% and recovery standard deviations from 18% to 31%. We did not adjust air concentrations for spike recoveries. Two laboratory technicians analyzed all PAH samples using the same technique. Measurement agreement of the duplicate PAH samples were > 90% over the 2-year exposure assessment period. The detection limit for each target PAH was 1.0 ± 0.2 ng/sample; 100% of the air samples were above the detection limit for all PAHs except for D(*a,h*)A (73% > detection limit). The study was reviewed and approved by the institutional review boards of Jagiellonian University and Columbia Presbyterian Medical Center. We obtained informed consent from all study participants.

### Statistical analyses

We limited statistical analysis to the air samples with a high/good quality assurance score (0 or 1): 489 (96%) personal air monitoring samples, 76 (97%) indoor, and 70 (91%) outdoor samples. Sixty-eight of 78 women completed all three serial personal monitorings and met the quality standard. The personal, indoor, and outdoor exposure levels of nine individual PAHs as well as ∑8c-PAHs (nanograms per cubic meter) were skewed (all *p*-values for Komolgorov–Smirnov test < 0.001). After natural log (ln) transformation, the distribution of the personal air samples remained bimodal, with a low exposure range during June–August and a high exposure range during September–May, whereas indoor and outdoor measurements conformed to a normal distribution. When stratified by season, all distributions approximated the normal distribution (Komolgorov–Smirnov tests *p* > 0.05). The PAH concentrations below the detection limit were assigned half of the limit value. We assessed agreement between repeated personal monitoring and simultaneous personal, indoor, and outdoor monitoring using Spearman’s rank correlation coefficients. Our statistical models used personal measurements of ln-transformed values for all nine PAHs as well as the ln-transformed ∑8c-PAH values as the outcomes. We developed a gestation-length model by hierarchically generalizing cross-sectional data of simultaneous personal, indoor, and outdoor monitoring data.

We first examined the personal exposure PAH levels as a function of the corresponding outdoor levels through ordinary least-squares regression. To apply this model to the remaining women who lacked outdoor and indoor monitoring data, we considered two approaches to estimate each woman’s outdoor concentration at a given gestational month. First, we estimated the outdoor concentration as the cohort’s outdoor mean level at a given month and year, ignoring the spatial variability. Whenever available, we used the actual data as the predictor of the personal exposure at a given gestational month. In the second approach, we considered the indicator variable for month and year of a given gestational month as a proxy for the outdoor concentration. In the resulting ordinary least-squares regression model of the personal exposure, we controlled for the effects of ETS exposure and residence in the city center. Given the result of this model, we considered repeated personal monitoring and indicator variables for month and year at a given gestational age as the main exposure variables in the random effects model. We used backward model selection strategy to reduce the number of fixed effect variables with selection criteria α = 0.10. However, we retained some variables in the final model regardless of this criterion, based on our prior knowledge regarding outdoor sources in Krakow. The final model included the fixed time (month and year indicator of personal monitoring, trimester at the time of monitoring), fixed spatial (living in city center), fixed behavior (hours of ETS exposure at home), and fixed interaction (December 2001, January 2002, and December and city center, respectively) terms, as well as the subject-specific random deviation from the population mean. We examined the precision of the predicted mean exposure at a given gestational month using the “leave-one-out” cross-validation method. We calculated the relative cross-validation residual as (predicted personal exposure concentration – observed personal exposure concentration)/observed personal exposure concentration. We fit the model with one observation deleted from the data at each time and used that observation as the test sample to estimate the prediction error. We conducted the statistical analyses in R version 2.5.1 (R Project for Statistical Computing, Vienna, Austria), SAS version 9.1 (SAS Institute Inc., Cary, NC, USA), and SPSS version 14.0 (SPSS Inc., Chicago, IL, USA).

## Results

### Environmental and behavior characteristics of the cohort

**Figure 1:**
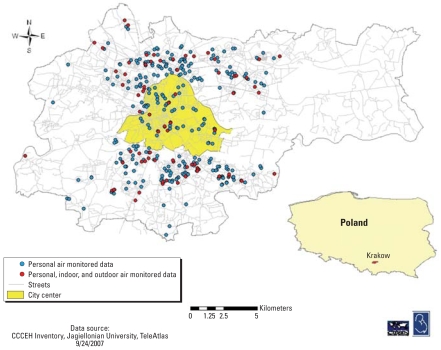
Home location of the pregnant women who completed personal and personal/indoor/outdoor monitoring. From Columbia Center for Children’s Environmental Health Inventory, Jagiellonian University, TeleAtlas, 24 September 2007.

The map of residential locations (Figure 1) shows that women lived in an area 20 × 20 km. We compared the demographic and exposure characteristics of the singly monitored women with the repeatedly monitored subset (Table 1). The goal of the comparison was to examine whether the exposure and behavior characteristics of the subset are representative for those with single personal exposure measurements. This would permit a merging of exposure data. All exposure and demographic characteristics were similar between the two groups, except that some of the repeatedly monitored women were more likely to spend > 10 hr/day at home. Most of the women reported that they spend ≤ 3 hr in the outdoor setting, 4–10 hr/day at home, and 1–2 hr/day commuting. Also, the reported amount of outdoor vehicular traffic intensity was “light” for most women in both groups. The frequency of dietary PAH exposure through consumption of grilled, barbecued, or blackened food items was low. Less than 5% of the subjects in either groups reported that they ate these items more than twice a week.

As the pregnancy progressed, personal behavior patterns changed. Among 286 women who initially reported no ETS exposure during the first and second trimester, 22 (8%) reported 1–4 hr/day of exposure, and 3 (1%) reported 5–10 hr/day during the third trimester (7% increase). Although 90% (*n* = 310) reported spending at least 5 hr/day at home during the second trimester, this increased to 98% (*n* = 333) during the third trimester.

Considering that the type of occupation has been shown to be associated with personal exposure levels of aromatic hydrocarbons (Ilgen et al. 2001), we examined whether this is also true with personal PAH exposure in this cohort. We also examined whether the number of hours spent outdoors, at home, or during commuting were associated with personal PAH exposure. Although a higher proportion of women who worked in restaurants reported that they spent > 3 hr outdoors (50% during April–September, 25% during October–March), the personal exposure concentration during the second trimester was not associated with the number of hours spent outdoors, at home, or in transit (all *p* > 0.05), nor was the type of occupation associated with the personal exposure level (*p* > 0.05).

### PAH concentrations based on personal, home indoor and home outdoor monitoring

Table 2 shows the concentration distributions of the nine PAHs and ∑8c-PAH according to the monitoring type and seasons of monitoring [see also Supplemental Material, Table 2 (online at http://www.ehponline.org/members/2008/10972/suppl.pdf)]. The nine PAHs and ∑8c-PAH for the personal, indoor, and outdoor levels (ng/m^3^) vary by more than 10-fold between winter (December–February) and summer (June–August). Mean personal exposure to ∑8c-PAH (ng/m^3^) during the winter is 60 ng/m^3^ higher than the mean during the summer (*p* < 0.001). Pyrene is the most abundant PAH, accounting for 20–26% of the total concentration during each season. B(*b*)F, B(*a*)P, B(*a*)A, and indeno(*1,2,3-c*,*d*)pyrene [I(*1*,*2*,*3*-*cd*)P] are the next most abundant PAHs, and their concentrations are comparable in all seasons. Personal, indoor, and outdoor PAH levels are virtually identical during the June–August period (mean personal – indoor difference = 0.01 ± 0.93 ng/m^3^; mean personal – outdoor difference = 0.75 ± 1.92 ng/m^3^). In contrast, the mean personal exposure concentration is higher than the home indoor level and lower than the home outdoor level (mean personal – indoor difference = 10.97 ± 30.66 ng/m^3^; mean personal – outdoor difference = 29.23 ± 28.07 ng/m^3^) between December and March (Jedrychowski et al. 2007).

High crude correlation coefficients between the second- and third-trimester personal monitoring values reflect the short temporal gap between the monitoring periods (mean, 6 weeks; range, 5–10 weeks) (Table 3). In contrast, the longer gap between the first and second personal monitoring (mean, 19 weeks; range, 17–23 weeks) yields lower crude correlation coefficients for the nine PAHs. Simultaneously monitored personal, indoor, and outdoor PAHs were highly correlated (pairwise Spearman’s coefficients for the nine PAHs ≥ 0.84, *p* < 0.01).

To identify the risk factors of personal exposure, we summarized mean personal PAH concentrations according to the potential risk factors [Table 4; see also Supplemental Material, Table 3 (online at http://www.ehponline.org/members/2008/10972/suppl.pdf)]. As expected, season was associated with large variation in personal exposure to the nine PAH levels and ∑8c-PAH (all *p*-values < 0.01). Compared with the nonsmokers, women who reported ≥ 5 hr/days of ETS were, on average, exposed to 0.95–7.48 ng/m^3^ higher concentrations for the nine PAHs (all *p*-values < 0.05). The mean difference for ∑8c-PAH for the same categories of women was 37 ng/m^3^ (*p* = 0.003). Neither the fuel type for home heating nor the outdoor traffic intensity was associated with significantly higher personal exposure. Women who lived near an industrial plant had significantly higher personal exposure to B(*a*)P, I(*1*,*2*,*3*-*cd*)P, and pyrene, as well as ∑8c-PAH (*p* < 0.05). For the women who spent > 3 hr/day outdoors, mean personal exposure to ∑8c-PAH was significantly lower (*p* = 0.011). However, contrary to our expectation, neither the duration of commute nor the time spent at home was associated with any change in personal exposure level. Despite no difference in commuting duration, women who commuted by tram had, on average, 13 ng/m^3^ higher personal exposure to ∑8c-PAH compared with those who walked (*p* < 0.01). In addition, those who used an exhaust fan during at least half of all cooking time were exposed to an 8 ng/m^3^ lower personal ∑8c-PAH concentration compared with those who did not use a fan (*p* = 0.017). To further clarify the role of using an exhaust fan, we repeated the comparison in season-stratified data, restricted to nonsmoker households. Although consistent exhaust fan users’ personal ∑8c-PAH was 10 and 14 ng/m^3^ lower during spring (*p* = 0.05) and winter (*p* > 0.10), respectively, the size of the reduction was < 3 ng/m^3^ during summer and fall (both *p*-values > 0.10).

### Short-term predictors of personal exposure to pyrene and ∑8c-PAH

We considered the following variables as potential risk factors of personal PAH exposure: residence in the city center, ambient temperature, wind speed, ETS exposure, frequency of exhaust fan use, residence near an industrial plant, commute by tram, apartment height (floor), home heating fuel of coal or wood, time spent outdoors (hour/day), and simultaneously monitored outdoor concentration. Table 5 shows personal exposure models for each PAH and includes only those variables that met our forward selection criterion (type 1 error = 0.10). During the heating season, a 1-ln-unit increase in the outdoor concentration of the individual PAHs and ∑8c-PAH was associated with a 91–100% increase in personal exposure. The efficiency of the outdoor concentration as a predictor precluded other variables. The negative *y*-intercept for most PAHs, although not significant, indicates that home concentrations were lower than the corresponding outdoor levels during the heating season.

In contrast, during the nonheating season, a 1-ln-unit increase in the outdoor concentration was associated with a 58–89% increase in personal exposure to the individual carcinogenic PAHs, despite the higher reported likelihood of window ventilation. Only during the nonheating season, ETS and ambient temperature significantly increase personal exposure to B(*b*)F, B(*g*,*h*,*i*)P, B(*a*)P, chrysene, I(*1*,*2*,*3*-*cd*)P, and ∑8c-PAH. A 1-hr increase in ETS exposure was associated with a 10–16% increase in personal exposure to these PAHs. Similarly, a 1°C decrease in ambient temperature was associated with a 3–5% increase in exposure for B(*a*)A, B(*k*)F, and D(*a*,*h*)A, after accounting for the outdoor concentration. The behavior of pyrene is unique, probably due to its low molecular weight. The distribution pattern and statistical association of the eight PAHs are consistent overall with those for ∑8c-PAH, demonstrating that ∑8c-PAH is an appropriate proxy of exposure to the eight individual carcinogenic PAHs.

### Estimated mean monthly exposure over the entire gestational period

**Figure 2:**
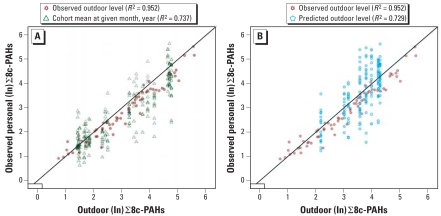
Estimation of personal (ln) ∑8c-PAH based on mean outdoor level (*A*) or indicator variable for given month and year (*B*). (*A*) Personal mean (ln) ∑8c-PAH at a given calendar month and year = α + β × (group’s mean outdoor level during given month and year) + SE. (*B*) Personal mean (ln) ∑8c-PAH at a given calendar month and year = α + β × (indicator variable for given month and year) + SE.

We examined the external generalizability of our outdoor monitoring data (Figure 2), considering those women with a single personal monitoring (*n* = 266) as the testing data set. In this group, we determined the fit of the model for personal exposure to ∑8c-PAH (dependent variable) as a function of either group mean outdoor PAH concentration for a given month and year (Figure 2A) or an indicator variable for the given month and year (Figure 2B). We compared the fit of the model for the testing data set with the model fit for the women with personal, indoor, and outdoor monitoring data (reference group). Although the fit of personal exposure based on the group’s mean outdoor level at given month and year (*R*^2^ = 0.74) or indicator variable of given month and year (*R*^2^ = 0.73) was lower than that for the reference group whose personal exposure level we simultaneously monitored with the outdoor level (*R*^2^ = 0.95), it is still considerably high (see Figure 2).

Additionally, we estimated individual exposure to ∑8c-PAH at a given gestational month as a function of the variables shown in Table 6. Compared with the initial model [Supplemental Material, Table 4 (online at http://www.ehponline.org/members/2008/10972/suppl.pdf)], we achieved a modest gain in model fit in the reduced model (Table 6). Compared with the second trimester, the exposure level of each subject during the third trimester is significantly lower, whereas that during the first trimester is not, adjusted for other covariates. A 1-hr increase in ETS exposure at home was associated with a 7% increase in personal exposure at a given gestational month. Because we suspect that there was an inversion of pollution in the city center during the winter of 2001–2002, we decided to account for the spatiotemporal interactions (December 2001, January 2002, and December and city center, respectively) despite the fact that our model failed to detect a significant increase during these periods.

**Figure 3:**
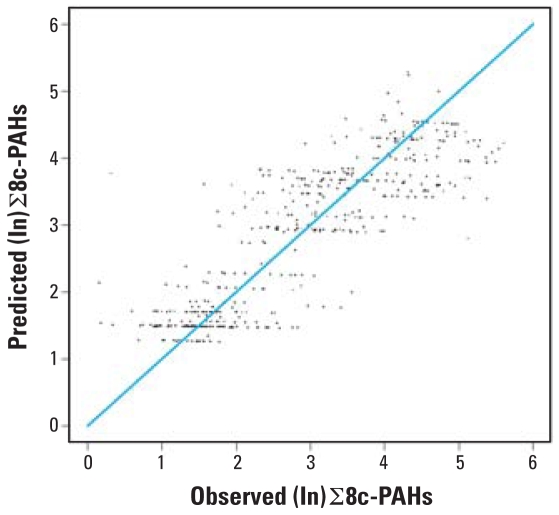
Predicted ∑8c-PAH exposure at sixth gestational month versus the observed ∑8c-PAH at same gestational period.

The range of predicted personal exposure to ∑8c-PAH was 3.42–151.41 ng/m^3^. In the random effects model (Table 6), all predictors are binary except for ETS exposure. As a result, the predicted personal exposures have wider range compared with the range of the predicted variables. The relative cross-validation residuals are clustered randomly around 0, indicating that our final model achieves good prediction (Figure 3). We also compared the prediction ability of our final model with a naive model based only on trimester and ETS exposure. The estimated prediction error of our final model is 0.81, whereas that of the naive model is 1.57, which demonstrates a much better prediction ability of our final model compared with the naive model.

## Discussion

Investigating health risks of PAH exposure, such as cancer or developmental effects, presents a number of challenges, the greatest of which is the lack of reliable and comprehensive human exposure data (Bostrom et al. 2002). Thus, the PAH monitoring scheme presented here with cross-sectional (simultaneous personal/indoor/outdoor monitoring) and a longitudinal component (repeated personal monitoring), supplemented by an in-depth questionnaire, provides airborne PAH exposure information for a nonoccupationally exposed population [Table 2; see also Supplemental Material, Table 2 (online at http://www.ehponline.org/members/2008/10972/suppl.pdf)].

Our first objective—to descriptively characterize the role of the home indoor and home outdoor setting on personal exposure of pregnant women—resulted in the following two conclusions: First, mean personal exposure during a given month and year is intermediate between indoor and outdoor levels during October–March. During June–August, mean personal exposure is almost identical to both the indoor and the outdoor level. A strong season-dependent rise in indoor PAH concentration, combined with the fact that most of the women spent most of their time at home, suggests that the home indoor environment offers little protection from outdoor PAHs, particularly during the heating season.

Second, the observed indoor and outdoor concentrations of B(*a*)P during spring/fall are comparable to the levels observed in other developed and developing countries. For example, the ambient B(*a*)P level in a heavily trafficked street in Copenhagen, Denmark, was 4.4 ± 1.2 ng/m^3^ in 1992 (Nielsen et al. 1996). Mean personal B(*a*)P exposure for traffic police officers in Beijing, China, was 51.9 ± 84.2 ng/m^3^ during winter (Liu et al. 2007). The mean indoor level in nonsmoker households during autumn in Hangzhou, China, was 4 ng/m^3^ (Liu et al. 2001). The mean ambient B(*a*)P level during the period of coal-fired power plant operation in Tongliang, China, was 16.8 ± 20.1 ng/m^3^ (Chow et al. 2006). In tropical cities such as Bangkok, Thailand, the median ambient level was 8.08 ng/m^3^ (Ruchirawa et al. 2002). At the same time, the Krakow B(*a*)P levels are higher than those in Los Angeles, California, (0.065 ng/m^3^), Houston, Texas, (0.025 ng/m^3^), and Elizabeth, New Jersey (0.14 ng/m^3^) (Naumova et al. 2002); Beauharnois, Quebec, Canada (0.177 ng/m^3^) (Sanderson and Farant 2004); Zagreb, Croatia (winter mean, 5.12 ± 3.46 ng/m^3^) (Sisovic et al. 2002), Grenoble, France (summer mean, 0.07 ± 0.02 ng/m^3^; winter mean, 1.02 ± 0.87 ng/m^3^) (Nielsen et al. 1996; Tao et al. 2006); and New York City (0.49 ± 0.65 ng/m^3^) (Choi et al. 2006). However, such international comparisons have limited health inferential value, not only because of the differences in PAH profiles, monitoring methodologies, monitoring durations, laboratory techniques, and instrument measurement errors, but also because of uncertainties in personal behavior choices and time spent in various microenvironmental settings. In the United States, the 10-min average PAH concentrations (nanograms per cubic meter) in various microenvironments, including food courts and buses, have been shown to have large inherent variability (Levy et al. 2002). Thus, short-term acute exposures in certain microenvironments, even in cities with documented low ambient PAH concentrations, might affect health outcomes, particularly in individuals with enhanced predisposition (Levy et al. 2002). Our present observation of lower exposure among the women who stay outdoors for longer hours is consistent with previous time–activity patterns of exposure to PM_2.5_ (Moschandreas and Saksena 2002). That is, microenvironments other than home are likely to provide greater protection among the women who report a higher proportion of daily hours in the nonhome setting.

Our second objective was to identify behavioral and environmental risk factors of personal PAH exposure during a given trimester using direct air monitoring and questionnaire data. Season-dependent outdoor sources are the most important risk factors of ∑8c-PAH exposure for the women in the study (Tables 4 and 5). This confirms a prior observation that coal burning for heating and industry is the main outdoor source of the ambient PAHs in Krakow (Jedrychowski et al. 2003). During the heating season, a 1-ln-unit increase in the outdoor concentration of the individual carcinogenic PAHs was associated with a 90–99% increase in the corresponding personal level. Outdoor (ln) ∑8c-PAH was associated with a 100% increase in the simultaneously monitored personal exposure level. The efficiency of the outdoor concentration as a predictor is high: The outdoor level alone accounts for 86–93% of total variability in personal exposure to each carcinogenic PAH. Contrary to our expectation, neither the indicators of spatial variability, including living near the bus depot, crossroad, or in the city center, nor the personal behavior choices, including time spent outdoors or commuting, were associated with higher personal exposure, after accounting for the ambient concentration. Considering that > 80% of the cohort spent < 3 hr/day outdoors, our analysis suggests that most women were exposed to outdoor-originating PAHs in the indoor setting. Although we did not assess the reliability for reporting time duration in specific locations, overall the time spent outdoors was strongly negatively correlated (Pearson’s correlation coefficient = −0.886, *p* < 0.001) with a derived total time spent in the indoor microenvironment (24 hr minus hours outdoors minus hours in transit). This provides some assurance that self-estimated time spent in general categories is reliable.

A 1ºC decrease in ambient temperature was associated with a 2% increase in personal (ln) ∑8c-PAH exposure during the April–September period, after accounting for the outdoor level. Although rigorous examination of the relationship between the concentration, ambient temperature, and pollutant chemistry is beyond the scope of this analysis, this suggests that the ambient temperature drop during early fall contributes to the increase in personal exposure concentration, independent of heating-related PAH generation. Also, only during the nonheating season, a 1-hr increase in home ETS exposure was associated with a 10% increase (95% confidence interval, 1–19%) in personal (ln) ∑8c-PAH.

We examined the within-Krakow generalizability of our cohort’s exposure data by fitting the group’s mean outdoor concentration on personal PAH concentration for the remaining women (*n* = 266) who lack outdoor measurement (Figure 2A). Overall, a high fit based on the outdoor concentration mean (*R*^2^ = 0.74) or the indicator variable for given month/year during 2001–2002 period (*R*^2^ = 0.73) demonstrates that either of these variables could be used to estimate mean personal exposure level for nonsmoking Krakow citizens. Currently, the Polish Environmental Protection Ministry does not conduct routine PAH monitoring in Krakow.

On the other hand, we developed a model of long-term individual levels of exposure (Table 6) specifically for the present cohort. We do not recommended application of the model to other segments of the Krakow population for several reasons. The women in the present study are not representative of the general Polish population. They are nonsmokers with high educational attainment and have no preexisting medical conditions. In addition, behavior choices of pregnant women are likely to be different from those of the general Krakow population. Furthermore, a combined contribution of Krakow’s heating sources (i.e., coal burning), industrial activity, geographic terrain, and meteorologic conditions limit the generalizability of the model for populations in other locations.

Accordingly, extension of the subset analysis (Table 5, Figure 2) to the entire cohort (Table 6) demonstrates that although short-term indoor levels reflect personal exposure levels more closely, PAH exposure for the duration of pregnancy is significantly influenced by the outdoor PAH level. This is consistent with prior observations of a strong influence of ambient PM concentrations on personal PM exposure levels (Kinney et al. 2006).

The mean individual level of exposure was significantly lower during the third trimester compared with the second trimester, controlling for the season, year, and ETS (Table 6). This implies that single 48-hr monitoring during the second trimester is not representative of exposure during the third trimester. Accordingly, we will examine intrauterine growth restriction based on the predicted exposure during all gestational months. During the data collection phase, we suspected that there was an air pollution inversion in the city center during early December. Thus, we retained second-order interaction terms, December 2001, January 2002, and December city center, to account for a brief period of acute exposure.

Because we did not monitor the PAH levels in microenvironments other than the home indoors, we cannot directly determine the relative proportion of total 48-hr personal PAH exposure associated with other microenvironments such as work, personal vehicle, or tram. Other limitations of the analysis include lack of information on building structure, ventilation frequency, and air exchange rate. Although coal-fired municipal furnaces and industrial activity are most likely to be the main source of the outdoor PAHs (Lvovsky et al. 2000), we could not determine the exact extent and duration of these sources during our air-monitoring campaign. Thus, we could not directly examine the relationship between specific sources and the personal exposure levels. Furthermore, we conceptualized season’s effect on PAH concentration as a proxy of ambient temperature (i.e., low temperature is related to personal exposure to PAHs through coal-fired furnaces). Season might have affected the exposure profile for each woman through other mechanisms, such as inversion. Also, we did not directly measure time–activity patterns for each person. Thus, the cumulative exposure over a 48-hr period could not be partitioned according to various microenvironments.

The U.K. government’s Expert Panel on Air Quality Standards has recommended an annual average standard for B(*a*)P of 0.25 ng/m^3^ (Dimashki et al. 2001). Although a similar recommendation does not exist for the United States, the Swedish guideline for B(*a*)P exposure is 0.1 ng/m^3^ (Bostrom et al. 2002). The personal, indoor, and outdoor B(*a*)P levels we observed here far exceeded the recommended level in all seasons and thus are of concern.

In summary, most women in the present cohort are exposed to outdoor-derived PAHs within the indoor setting. That is, indoor concentration is a better predictor of short-term (i.e., 48-hr period) personal exposure; however, long-term personal exposure is largely determined by the outdoor PAH concentration. Mean personal exposure at a given gestational month depends on the season at a given gestational age. Unique demographic attributes of the present cohort and environmental conditions of Krakow mean that personal exposure assessment through a cross-sectional and longitudinal monitoring scheme, supplemented by questionnaire, are critical for a comprehensive understanding of individual-level exposure. Furthermore, the overwhelming influence of the outdoor level on personal exposure means that general Krakow population exposure can be estimated with fairly high precision based on the outdoor mean concentration at given month/year. However, because of the specific behavior patterns of pregnant women, the estimated individual level of exposure based on a random effects model (Table 6) may not be generalizable to the unmeasured Krakow population.

## Figures and Tables

**Table 1:** Demographic and exposure characteristics of singly monitored versus personal/indoor/outdoor-monitored subjects [mean ± SD (minimum–maximum) or no. (%)].

Characteristic	Single personal measurement^a^ (*n* = 266)	Personal/indoor/outdoor measurement^b^ (*n* = 78)	*p*-Value
Mother’s age (years)	28 ± 3 (18–36)	28 ± 4 (18–35)	0.41
Prepregnancy weight (kg)	58.04 ± 8.20 (40–90)	59.94 ± 11.43 (43–118)	0.11
Mother’s height (cm)	165.08 ± 5.54 (149–180)	164.69 ± 5.92 (144–178)	0.56
Annual mean household income^c^ [no. (%)]			0.72
Low	178 (67)	54 (69)	
Medium	13 (5)	3 (4)	
High	4 (1)	0 (0)	
Refused/don’t know	71 (27)	21(27)	
Maternal education [no. (%)]			0.96
< High school	28 (10)	9 (11)	
High school graduate	71 (27)	20 (26)	
> High school	167 (63)	49 (63)	
Occupation during current pregnancy [no. (%)]			0.68
Office setting^d^	152 (57)	45 (58)	
Restaurant/factory	14 (5)	6 (8)	
Other/unknown	100 (38)	27 (35)	
Residence area^e^ [no. (%)]			0.46
Outer area (low pollution)	208 (78)	64 (82)	
City center (high pollution)	58 (22)	14 (18)	
Prior live birth [no. (%)]	96 (36)	30 (39)	0.70
Currently married [no. (%)]	247 (93)	73 (94)	0.82
Wine, beer, liquor glasses/day [no. (%)]	3 (1)	1 (1)	0.91
Daily home ETS exposure [no. (%)]			0.67
≤4 hr/day	36 (14)	10 (13)	
≥5 hr/day	8 (3)	4 (5)	
Nonsmoker home	222 (84)	64 (82)	
Time spent outdoor ≤3 hr/day [no. (%)]	209 (79)	64 (82)	0.50
Time spent at home [no. (%)]			< 0.01
≤3 hr/day	26 (10)	8 (10)	
4–10 hr/day	218 (82)	53 (68)	
10–16 hr/day	22 (8)	17 (22)	
Time spent in transit [no. (%)]			0.19
< 1 hr/day	7 (3)	4 (5)	
1–2 hr/day	221 (83)	68 (87)	
≥3 hr/day	38 (14)	6 (8)	
Amount of outdoor vehicular traffic [no. (%)]			0.12
Light	216 (81)	56 (72)	
Medium	37 (14)	14 (18)	
Heavy	13 (5)	8 (10)	
Live next door to outdoor sources [no. (%)]^f^			
Repair garage	46 (17)	17 (22)	0.37
Industrial plant	11 (4)	4 (5)	0.71
Bus depot	27 (10)	8 (10)	0.98
Incinerator	2 (1)	0 (0)	0.44
Fuel type^g^ [no. (%)]			0.16
Gas	60 (2)	14 (18)	
Electricity	26 (10)	15 (19)	
Coal	15 (6)	3 (4)	
Wood	4 (2)	0 (0)	
Town central heating	152 (57)	46 (59)	
Heating method [no. (%)]			0.25
Radiator	237 (89)	64 (82)	
Stove	10 (4)	3 (4)	
Electric heater	12 (5)	8 (10)	
Other	7 (3)	3 (4)	
Commuting method [no. (%)]			0.43
Tram	47 (18)	12 (15)	
Bus	49 (18)	22 (28)	
Drive/use taxi	97 (37)	26 (33)	
Walk/bike	72 (27)	18 (23)	
Exhaust fan use [no. (%)]			0.19
Never/no fan	132 (50)	45 (58)	
Sometimes	50 (19)	8 (10)	
≥Half time	84 (32)	25 (32)	
Burn candle at home (yes) [no. (%)]	177 (67)	45 (58)	0.15
Burn incense at home (yes) [no. (%)]	61 (23)	18 (23)	0.98

a Subject was monitored only for personal exposure during late second to early third trimester.

b Subject was monitored for personal exposure for a 48-hr period during each trimester, in addition to indoor and outdoor monitoring using an identical instrument.

c Low, < 37,024 Poland zlotych (PLZ); medium, 37,024–74,048 PLZ; high > 74,048 PLZ. One Euro = 4.2009 PLZ in 2002.

d Sales/telemarketing/school/health care/office work.

e Enrollment target sites were chosen based on 1996 air monitoring data from the U.S. EPA (U.S. EPA 1999), which showed that B(*a*)P was 2-fold higher in the city center than the outer area.

f Multiple sources are identified for each person. Thus, sources were treated as a separate question.

g Eight women (3%) from singly monitored group did not report their fuel type.

**Table 2:** Personal exposure, indoor, and outdoor levels (ng/m^3^), according to the season^a^ at time of monitoring.

	March–May					June–August					September–November					December–February				
	No.	GM	GSD	Min	Max	No.	GM	GSD	Min	Max	No.	GM	GSD	Min	Max	No.	GM	GSD	Min	Max
B(*a*)A																				
Personal, 1st trimester	18	2.02	3.11	0.25	10.38	23	0.45	1.46	0.24	1.06	16	0.45	1.46	0.09	43.14	19	8.62	2.87	0.42	35.49
Personal, 2nd trimester	86	2.28	2.95	0.29	39.69	81	0.57	1.57	0.23	3.05	82	2.50	2.83	0.35	26.24	92	9.91	2.18	1.29	33.83
Personal, 3rd trimester	20	1.38	2.64	0.40	12.95	18	0.45	1.20	0.30	0.63	18	2.04	1.86	0.41	5.10	16	5.59	2.53	1.17	27.47
Indoor	21	2.47	2.75	0.41	22.42	18	0.56	1.67	0.21	1.50	19	1.57	2.46	0.33	8.95	16	7.31	2.63	1.37	28.25
Outdoor	21	3.74	3.16	0.47	55.39	16	0.56	1.71	0.22	1.86	17	2.71	2.97	0.31	24.39	14	14.43	2.37	2.57	38.01
B(*b*)F																				
Personal, 1st trimester	18	3.10	2.64	0.53	15.09	23	1.14	1.60	0.40	3.49	16	1.14	1.60	0.29	42.28	19	8.41	3.62	0.09	32.23
Personal, 2nd trimester	86	3.49	2.75	0.52	45.67	81	1.13	1.70	0.26	8.39	82	4.85	2.64	0.67	55.57	92	14.15	2.04	2.36	67.47
Personal, 3rd trimester	20	1.88	2.71	0.51	26.75	18	0.58	1.84	0.18	1.20	18	3.87	1.93	0.57	7.32	16	9.41	2.60	1.25	35.76
Indoor	21	3.66	2.20	0.97	20.95	18	0.99	1.76	0.43	3.31	19	3.30	2.26	0.70	15.96	16	10.84	2.37	2.13	31.27
Outdoor	21	5.92	2.35	1.16	38.32	16	1.14	1.78	0.44	3.27	17	5.18	2.43	0.75	28.14	14	16.96	2.19	3.66	55.75
B(*k*)F																				
Personal, 1st trimester	18	1.14	3.07	0.09	6.52	23	0.38	1.83	0.09	1.47	16	0.38	1.83	0.09	15.35	19	3.01	2.95	0.09	10.50
Personal, 2nd trimester	86	1.13	2.92	0.09	13.04	81	0.40	1.82	0.09	3.19	82	1.45	2.53	0.27	17.40	92	4.59	2.10	0.61	20.33
Personal, 3rd trimester	20	0.70	2.78	0.23	6.81	18	0.19	1.87	0.09	0.50	18	1.13	1.91	0.20	2.50	16	3.50	2.41	0.88	24.06
Indoor	21	1.14	2.34	0.26	6.91	18	0.36	1.74	0.09	1.06	19	1.01	2.27	0.09	3.98	16	3.28	2.32	0.61	10.90
Outdoor	21	1.57	2.95	0.09	13.11	16	0.37	1.82	0.09	1.24	17	1.46	2.63	0.09	6.07	14	5.34	2.15	1.11	16.20
B(*g*,*h*,*i*)P																				
Personal, 1st trimester	18	2.09	2.75	0.40	12.49	23	0.62	1.57	0.28	2.09	16	0.62	1.57	0.21	26.51	19	5.88	3.50	0.09	26.55
Personal, 2nd trimester	86	2.15	2.87	0.24	32.26	81	0.71	1.59	0.22	2.28	82	2.77	2.53	0.49	32.61	92	6.96	2.01	1.14	30.23
Personal, 3rd trimester	20	1.32	2.70	0.37	15.93	18	0.41	1.88	0.09	0.76	18	2.22	1.80	0.42	4.24	16	4.78	2.35	0.98	18.62
Indoor	21	2.40	2.64	0.44	20.03	18	0.68	1.67	0.24	1.41	19	1.86	2.20	0.37	7.73	16	5.21	2.33	1.09	19.44
Outdoor	21	3.05	2.74	0.51	29.42	16	0.71	1.85	0.27	1.43	17	2.33	2.41	0.36	11.03	14	6.86	2.27	1.44	29.14
B(*a*)P																				
Personal, 1st trimester	18	2.11	3.38	0.25	16.30	23	0.47	1.58	0.21	1.56	16	0.47	1.58	0.09	53.71	19	7.21	3.56	0.09	30.68
Personal, 2nd trimester	86	2.38	3.14	0.22	37.40	81	0.54	1.74	0.09	1.80	82	2.99	2.97	0.36	33.34	92	10.66	2.18	1.16	42.23
Personal, 3rd trimester	20	1.52	2.83	0.35	20.41	18	0.42	1.28	0.26	0.70	18	2.47	1.96	0.38	5.29	16	6.01	2.41	1.09	22.67
Indoor	21	2.54	2.67	0.41	22.97	18	0.46	1.94	0.09	1.41	19	1.97	2.44	0.32	11.88	16	7.35	2.58	1.37	25.52
Outdoor	21	3.05	2.71	0.44	29.42	16	0.46	2.03	0.09	1.70	17	2.36	2.79	0.29	18.93	14	9.89	2.36	1.91	33.12
Chrysene																				
Personal, 1st trimester	18	1.88	2.56	0.28	7.07	23	0.58	1.58	0.26	1.50	16	0.58	1.58	0.09	32.92	19	6.24	2.58	0.43	22.93
Personal, 2nd trimester	86	2.07	2.76	0.32	30.31	81	0.66	1.64	0.19	3.76	82	2.36	2.78	0.27	23.50	92	8.02	2.10	1.12	31.13
Personal, 3rd trimester	20	1.12	2.54	0.31	10.01	18	0.36	1.92	0.09	0.77	18	1.90	1.96	0.28	3.96	16	4.72	2.71	0.95	24.15
Indoor	21	2.22	2.56	0.43	17.00	18	0.62	1.69	0.28	1.90	19	1.52	2.46	0.27	7.29	16	5.79	2.55	1.27	20.08
Outdoor	21	4.17	2.68	0.64	38.13	16	0.76	1.68	0.37	2.60	17	3.28	2.63	0.42	19.44	14	11.86	2.25	2.79	37.47
D(*a*,*h*)A																				
Personal, 1st trimester	18	0.43	3.14	0.09	2.87	23	0.10	1.36	0.08	0.27	16	0.10	1.36	0.09	6.27	19	1.55	2.88	0.09	8.08
Personal, 2nd trimester	86	0.44	3.21	0.08	9.41	81	0.12	1.62	0.08	0.40	82	0.54	2.92	0.09	7.38	92	1.66	2.12	0.23	10.76
Personal, 3rd trimester	20	0.24	3.14	0.09	4.36	18	0.10	1.31	0.08	0.23	18	0.46	1.78	0.09	0.83	16	1.14	2.37	0.22	4.22
Indoor	21	0.54	3.16	0.08	5.79	18	0.14	1.81	0.09	0.39	19	0.38	2.20	0.09	1.89	16	1.26	2.55	0.24	4.79
Outdoor	21	0.68	3.29	0.08	8.15	16	0.16	1.87	0.09	0.41	17	0.50	2.44	0.09	2.92	14	1.70	2.34	0.33	6.93
I(1,2,3-*c*,*d*)P																				
Personal, 1st trimester	18	2.38	3.05	0.38	15.36	23	0.63	1.55	0.28	1.81	16	0.63	1.55	0.23	33.79	19	7.66	3.73	0.09	36.06
Personal, 2nd trimester	86	2.71	3.04	0.26	50.20	81	0.74	1.56	0.25	1.97	82	3.15	2.68	0.44	36.26	92	8.95	2.03	1.48	39.58
Personal, 3rd trimester	20	1.54	2.85	0.38	23.05	18	0.46	1.73	0.09	0.82	18	2.56	1.84	0.54	5.35	16	5.74	2.35	1.04	22.30
Indoor	21	3.24	2.78	0.45	32.71	18	0.75	1.71	0.29	1.62	19	2.12	2.28	0.38	11.30	16	6.66	2.36	1.43	22.05
Outdoor	21	4.00	2.74	0.58	31.42	16	0.80	1.91	0.30	1.87	17	2.73	2.52	0.37	16.49	14	9.04	2.24	1.95	34.03
Pyrene																				
Personal, 1st trimester	18	4.56	2.24	1.18	19.65	23	1.47	1.41	0.83	2.94	16	1.47	1.41	0.26	33.05	19	12.46	2.21	3.64	44.30
Personal, 2nd trimester	86	4.43	2.27	0.80	47.12	81	2.22	1.64	1.04	21.45	82	4.15	2.29	0.95	32.49	92	14.10	2.16	1.78	61.96
Personal, 3rd trimester	20	3.54	2.01	1.07	12.79	18	1.95	1.62	0.68	4.87	18	3.42	1.51	1.22	7.11	16	8.56	2.37	2.62	39.26
Indoor	21	4.14	2.32	1.10	31.06	18	1.86	1.39	1.09	3.47	19	2.56	2.04	0.73	9.80	16	9.24	2.35	1.95	37.85
Outdoor	21	7.65	2.51	1.59	78.99	16	2.38	1.62	1.13	4.34	17	6.14	2.27	1.28	27.80	14	21.55	2.25	4.44	61.00
∑8c-PAH																				
Personal, 1st trimester	18	15.36	2.87	2.52	82.55	23	4.45	1.52	1.99	12.74	16	4.45	1.52	1.16	253.44	19	49.99	3.05	1.37	196.81
Personal, 2nd trimester	86	16.88	2.88	2.03	241.76	81	5.02	1.56	1.81	16.92	82	20.84	2.68	3.38	232.30	92	65.61	2.06	10.15	272.18
Personal, 3rd trimester	20	9.80	2.70	2.62	120.29	18	3.09	1.50	1.19	5.16	18	16.75	1.87	2.87	33.17	16	41.96	2.38	10.61	166.84
Indoor	21	18.46	2.54	3.45	148.80	18	4.68	1.64	2.04	11.92	19	13.88	2.28	2.59	68.87	16	48.04	2.44	9.49	149.71
Outdoor	21	26.52	2.69	4.12	243.35	16	5.07	1.75	2.02	13.81	17	20.74	2.57	2.69	127.41	14	76.59	2.25	15.76	250.64

Abbreviations: GM, geometric mean; GSD, geometric standard deviation; Max, maximum; Min, minimum.

a Season is determined at the time of monitoring, so season for those persons with serial monitoring refers to different calendar periods.

**Table 3:** Crude Spearman correlation coefficients of repeated personal measurements and contemporaneous personal/indoor/outdoor measurements (*n* = 68).

	Across repeated personal monitoring		Simultaneous personal/indoor/outdoor monitoring		
PAH	2nd vs. 1st monitoring	2nd vs. 3rd monitoring*	2nd personal vs. indoor*	2nd personal vs. outdoor*	2nd indoor vs. outdoor*
B(*a*)A	0.19	0.54	0.96	0.98	0.97
B(*b*)F	0.08	0.48	0.97	0.97	0.97
B(*k*)F	0.05	0.62	0.95	0.96	0.96
B(*g*,*h*,*i*)P	0.15	0.44	0.95	0.97	0.97
B(*a*)P	0.10	0.57	0.95	0.97	0.97
Chrysene	0.13	0.49	0.96	0.96	0.96
D(*a*,*h*)A	0.23	0.41	0.95	0.96	0.96
I(*1,2,3-c*,*d*)P	0.19	0.43	0.96	0.97	0.98
Pyrene	0.11	0.53	0.86	0.84	0.93
∑8c-PAH	0.12	0.51	0.96	0.98	0.97
Days between monitoring [mean ± SD (Min–Max)]	88 ± 6 (65–105)	47 ± 7 (33–72)	—	—	—

Abbreviations: Max, maximum; Min, minimum.

* *p* < 0.01 for all values, based on pairwise Spearman rank correlation.

**Table 4:** Personal exposure concentration (geometric mean and 95% confidence interval) to individual PAHs (ng/m^3^) for the pregnant women.

Factor	B(*a*)A	B(*b*)F	B(*k*)F	B(*g*,*h*,*i*)P	B(*a*)P	Chrysene	D(*a*,*h*)A	I(1,2,3-*c*,*d*)P	Pyrene
Season^a^									
April–September (*n* = 160)	0.82 (0.73–0.92)	1.57 (1.4–1.76)	0.52 (0.47–0.59)	0.96 (0.86–1.07)	0.83 (0.73–0.95)	0.88 (0.79–0.98)	0.17 (0.15–0.19)	1.05 (0.93–1.18)	2.41 (2.22–2.62)
October–March (*n* = 181)	6.65** (5.8–7.61)	10.12** (8.92–11.47)	3.21** (2.83–3.65)	5.45** (4.85–6.13)	7.33** (6.4–8.41)	5.61** (4.92–6.39)	1.24** (1.09–1.41)	6.86** (6.08–7.75)	9.71** (8.55–11.02)
Residence									
Outer city area (*n* = 270)	2.28 (1.94–2.66)	3.88 (3.36–4.48)	1.26 (1.09–1.45)	2.22 (1.94–2.54)	2.39 (2.03–2.83)	2.16 (1.87–2.50)	0.45 (0.39–0.53)	2.62 (2.26–3.02)	4.67 (4.16–5.25)
City center (*n* = 71)	3.54 (2.54–4.94)	5.8 (4.29–7.85)	1.91 (1.42–2.58)	3.32 (2.51–4.4)	3.85 (2.73–5.44)	3.2 (2.36–4.34)	0.65 (0.47–0.9)	3.92 (2.91–5.28)	6.82 (5.31–8.76)
ETS(hr/day)									
Nonsmoker (*n* = 283)	2.51 (2.16–2.93)	4.24 (3.68–4.89)	1.38 (1.2–1.59)	2.41 (2.11–2.76)	2.66 (2.27–3.13)	2.34 (2.03–2.69)	0.49 (0.42–0.57)	2.86 (2.48–3.29)	5.00 (4.46–5.62)
< 5 hr/day (*n* = 46)	1.75 (1.17–2.63)	3.14 (2.19–4.49)	0.98 (0.68–1.4)	1.87 (1.35–2.58)	1.86 (1.21–2.88)	1.79 (1.23–2.59)	0.36 (0.25–0.52)	2.12 (1.48–3.02)	4.25 (3.18–5.66)
≥5 hr/day (*n* = 12)	8.13* (3.46–19.09)	11.37* (5.58–23.16)	4.05* (1.92–8.58)	6.27* (2.95–13.32)	8.51* (3.66–19.78)	7.12* (3.52–14.38)	1.44* (0.61–3.38)	8.05* (3.72–17.44)	12.48* (6.32–24.65)
Coal/wood heating									
No (*n* = 319)	2.48 (2.14–2.88)	4.2 (3.66–4.81)	1.37 (1.2–1.56)	2.4 (2.12–2.73)	2.63 (2.26–3.07)	2.34 (2.04–2.68)	0.49 (0.42–0.56)	2.84 (2.48–3.25)	5.06 (4.53–5.65)
Yes (*n* = 22)	2.68 (1.51–4.78)	4.55 (2.69–7.7)	1.42 (0.79–2.54)	2.53 (1.55–4.13)	2.8 (1.45–5.39)	2.41 (1.42–4.09)	0.53 (0.31–0.89)	2.97 (1.77–4.99)	4.98 (3.25–7.64)
Outdoor traffic									
Light (*n* = 84)	2.94 (2.18–3.96)	4.85 (3.67–6.39)	1.56 (1.17–2.07)	2.77 (2.15–3.57)	3.13 (2.29–4.29)	2.76 (2.09–3.63)	0.55 (0.41–0.73)	3.25 (2.49–4.25)	5.78 (4.59–7.27)
Medium (*n* = 51)	3.00 (2.06–4.37)	5.03 (3.56–7.1)	1.6 (1.14–2.25)	2.92 (2.12–4.03)	3.31 (2.28–4.83)	2.74 (1.93–3.88)	0.56 (0.38–0.83)	3.48 (2.47–4.91)	5.60 (4.2–7.45)
Heavy (*n* = 20)	3.52 (1.82–6.81)	5.84 (3.16–10.79)	1.73 (0.91–3.29)	3.49 (1.84–6.61)	3.65 (1.79–7.43)	3.25 (1.77–5.94)	0.73 (0.35–1.51)	4.06 (2.04–8.07)	6.27 (3.72–10.56)
Industrial plant									
Yes (*n* = 15)	6.36* (3.05–13.29)	8.99 (4.58–17.62)	2.59 (1.27–5.26)	5.03 (2.62–9.67)	6.65* (3.11–14.26)	5.19 (2.55–10.56)	1.09 (0.52–2.31)	6.41* (3.21–12.82)	10.06* (5.77–17.53)
No (*n* = 326)	2.39 (2.07–2.76)	4.07 (3.57–4.65)	1.33 (1.17–1.52)	2.33 (2.06–2.64)	2.53 (2.18–2.95)	2.26 (1.98–2.58)	0.47 (0.41–0.54)	2.74 (2.4–3.13)	4.9 (4.39–5.46)
Bus depot									
Yes (*n* = 35)	1.94 (1.22–3.06)	3.35 (2.17–5.17)	1.05 (0.67–1.64)	1.84 (1.22–2.77)	2.04 (1.25–3.32)	1.94 (1.27–2.95)	0.36 (0.22–0.57)	2.17 (1.39–3.39)	4.27 (3.04–5.99)
No (*n* = 306)	2.57 (2.21–2.99)	4.33 (3.77–4.97)	1.41 (1.24–1.62)	2.49 (2.19–2.83)	2.72 (2.33–3.19)	2.4 (2.09–2.75)	0.51 (0.44–0.58)	2.93 (2.56–3.37)	5.15 (4.6–5.77)
Live near crossroad									
Yes (*n* = 256)	2.39 (2.03–2.82)	4.08 (3.51–4.73)	1.33 (1.14–1.54)	2.31 (2–2.66)	2.51 (2.11–2.98)	2.28 (1.96–2.65)	0.47 (0.4–0.56)	2.74 (2.35–3.18)	4.89 (4.33–5.53)
No (*n* = 85)	2.83 (2.1–3.81)	4.67 (3.55–6.14)	1.51 (1.16–1.98)	2.75 (2.14–3.53)	3.1 (2.28–4.22)	2.56 (1.95–3.37)	0.53 (0.4–0.71)	3.2 (2.45–4.18)	5.58 (4.46–6.97)
Total outdoor time									
≤ 3 hr/day (*n* = 271)	2.74 (2.33–3.21)	4.6 (3.98–5.32)	1.51 (1.30–1.74)	2.62 (2.28–3.00)	2.9 (2.46–3.43)	2.55 (2.2–2.95)	0.53 (0.45–0.62)	3.09 (2.67–3.57)	5.43 (4.82–6.12)
> 3 hr/day (*n* = 70)	1.75 (1.28–2.39)	3.01 (2.26–4.01)	0.96 (0.71–1.28)	1.75 (1.33–2.31)	1.84 (1.32–2.57)	1.71 (1.28–2.27)	0.35 (0.26–0.47)	2.08 (1.55–2.78)	3.83 (3.03–4.84)
Commute time									
≤ 2 hr/day (*n* = 297)	2.57 (2.21–2.99)	4.32 (3.76–4.97)	1.4 (1.22–1.61)	2.48 (2.17–2.82)	2.71 (2.31–3.19)	2.4 (2.09–2.76)	0.5 (0.44–0.58)	2.92 (2.53–3.35)	5.2 (4.64–5.83)
> 2 hr/day (*n* = 44)	2.05 (1.36–3.08)	3.56 (2.4–5.28)	1.17 (0.8–1.72)	2.02 (1.41–2.88)	2.23 (1.46–3.4)	2.01 (1.35–2.97)	0.39 (0.26–0.59)	2.41 (1.65–3.54)	4.16 (3.08–5.62)

a Season was determined at the time of monitoring, so season for those persons with serial monitoring refers to different calendar periods.

* *p* < 0.05;

** *p* < 0.01.

**Table 5:** Risk factors of personal exposure to PAHs, based on simultaneously monitored levels and other putative predictors.

Outcome: Personal exposure to PAH	Heating season (October–March)				Nonheating season (April–September)			
	Slope	95% CI	*R*^2^	Δ*R*^2^	Slope	95% CI	*R*^2^	Δ*R*^2^
(ln)B(*a*)A								
*y-*Intercept	−0.30	−0.62 to 0.03			0.21	−0.19 to 0.60		
Outdoor (ln)B(*a*)A	0.92	0.79 to 1.05	0.897	0.897	0.66	0.55 to 0.78	0.914	0.914
Temperature (°C)					−0.03	−0.05 to 0.00	0.929	0.015
(ln)B(*b*)F								
*y-*Intercept	−0.23	−0.52 to 0.06			−0.23	−0.36 to −0.09		
Outdoor (ln)B(*b*)F	0.97	0.85 to 1.08	0.931	0.931	0.82	0.72 to 0.92	0.917	0.917
ETS (hr)					0.10	0.01 to 0.20	0.933	0.016
(ln)B(*k*)F								
*y-*Intercept	−0.17	−0.40 to 0.07			0.33	−0.13 to 0.80		
Outdoor (ln)B(*k*)F	0.91	0.76 to 1.06	0.868	0.868	0.58	0.42 to 0.74	0.826	0.826
Temperature (°C)					−0.05	−0.09 to −0.02	0.881	0.055
(ln)B(*g*,*h*,*i*)P								
*y-*Intercept	−0.11	−0.37 to 0.14			0.22	−0.16 to 0.60		
Outdoor (ln)B(*g*,*h*,*i*)P	0.98	0.86 to 1.11	0.928	0.928	0.78	0.67 to 0.89	0.909	0.909
Temperature (°C)	0.02	0.00 to 0.04	0.941	0.012	−0.03	−0.05 to 0.00	0.936	0.027
ETS (hr)					0.12	0.04 to 0.21	0.950	0.015
Apt height (floor)					0.17	0.00 to 0.35	0.960	0.010
(ln)B(*a*)P								
*y-*Intercept	−0.05	−0.31 to 0.20			−0.13	−0.26 to 0.01		
Outdoor (ln)B(*a*)P	0.96	0.84 to 1.08	0.921	0.921	0.89	0.77 to 1.01	0.904	0.904
ETS (hr)					0.14	0.02 to 0.26	0.925	0.021
(ln)Chrysene								
*y-*Intercept	−0.54	−0.83 to −0.25			−0.42	−0.54 to −0.31		
Outdoor (ln)chrysene	0.99	0.87 to 1.12	0.917	0.917	0.74	0.63 to 0.84	0.885	0.885
ETS (hr)					0.13	0.03 to 0.22	0.915	0.030
(ln)D(*a*,*h*)A								
*y-*Intercept	−0.14	−0.24 to −0.04			0.04	−0.45 to 0.53		
Outdoor (ln)D(*a*,*h*)A	1.00	0.89 to 1.11	0.938	0.938	0.80	0.62 to 0.98	0.874	0.874
Temperature (°C)					−0.04	−0.08 to 0.00	0.893	0.019
(ln)I(1,2,3-*c*,*d*)P								
*y-*Intercept	−0.27	−0.58 to 0.04			−0.23	−0.39 to −0.08		
Outdoor (ln)I(1,2,3-*c*,*d*)P	1.03	0.90 to 1.17	0.914	0.914	0.85	0.75 to 0.95	0.906	0.906
ETS (hr)	0.07	0.00 to 0.14	0.928	0.015	0.16	0.06 to 0.25	0.930	0.024
Apt height (floor)					0.26	0.06 to 0.46	0.950	0.020
Temperature (°C)	0.02	0.00 to 0.04	0.940	0.012				
(ln)Pyrene								
*y-*Intercept	−0.30	−0.74 to 0.14			0.08	−0.14 to 0.31		
Outdoor (ln)pyrene	0.87	0.72 to 1.02	0.855	0.855	0.60	0.45 to 0.75	0.733	0.733
Commute by tram					0.29	0.01 to 0.57	0.783	0.049
(ln)∑8c-PAH								
(Constant)	−0.41	−1.01 to 0.19			0.53	−0.02 to 1.09		
Outdoor (ln)∑8c-PAH	1.02	0.88 to 1.16	0.925	0.925	0.75	0.64 to 0.86	0.921	0.921
Temperature (°C)	0.02	−0.00 to 0.04	0.934	0.009	−0.02	−0.05 to 0.00	0.939	0.017
ETS (hr)					0.10	0.01 to 0.19	0.949	0.010
Apt height (floor)					0.16	−0.02 to 0.34	0.957	0.008

Abbreviations: Apt, apartment; CI, confidence interval. We forward-selected the model in stepwise model selection strategy, with α = 0.10, from a pool of potential predictors: outdoor concentration of given PAH, residence location (city center = 1, outer area = 0), ambient temperature, wind speed, ETS exposure, frequency of exhaust fan use, live near an industrial plant, commute by tram, apartment height (floor), home heating with fuel of coal or wood, time spent outdoors (hr/day).

**Table 6:** Random-effects model^a^ of mean personal exposure to (ln)∑8c-PAH at a given gestational month.

Factor	Slope	SE	*p*-Value
*y*-Intercept	2.3909	0.2580	< 0.0001
Gestational age			
Second trimester	Reference		
First trimester	−0.1467	0.1081	0.1756
Third trimester	−0.4220	0.1072	0.0001
Household member behavior			
ETS at home (hr)	0.0677	0.0293	0.0214
Residence location			
Outer area	Reference		
City center	0.0707	0.0915	0.4402
Personal monitoring period			
May–August	Reference		
September	0.4418	0.1210	0.0003
October	1.3573	0.1259	< 0.0001
November	1.7547	0.1587	< 0.0001
December	1.8279	0.4962	0.0003
January	2.1199	0.1999	< 0.0001
February	2.0851	0.1327	< 0.0001
March	1.6439	0.1265	< 0.0001
April	1.2639	0.1241	< 0.0001
Year 2000	Reference		
Year 2001	−0.5826	0.2235	0.0096
Year 2002	−0.8216	0.2245	0.0003
Interaction terms			
December 2001	0.0366	0.3192	0.9087
January 2002	0.3416	0.2468	0.1674
December and city center	0.1599	0.3840	0.6774

a The variables listed here are fixed factors, and we considered the person effect a random factor. We did not consider temperature and wind speed as confounders because of high missing values.
